# Vaccine Attitudes and COVID-19 Vaccine Intentions and Prevention Behaviors among Young People At-Risk for and Living with HIV in Los Angeles and New Orleans

**DOI:** 10.3390/vaccines10030413

**Published:** 2022-03-09

**Authors:** Dallas Swendeman, Peter Norwood, Jessica Saleska, Katherine Lewis, Wilson Ramos, Nicholas SantaBarbara, Stephanie Sumstine, Warren Scott Comulada, Sergio Jimenez, Manuel A. Ocasio, Elizabeth M. Arnold, Karin Nielsen-Saines, Maria Isabel Fernandez, Mary Jane Rotheram-Borus, on behalf of the Adolescent HIV Medicine Trials Network (ATN) CARES Team

**Affiliations:** 1 Center for Community Health, Department of Psychiatry and Biobehavioral Sciences, David Geffen School of Medicine, University of California Los Angeles, Los Angeles, CA 90024, USA; pnorwood@mednet.ucla.edu (P.N.); jess.saleska@gmail.com (J.S.); katlew@ucla.edu (K.L.); wramos@mednet.ucla.edu (W.R.); ssumstine@mednet.ucla.edu (S.S.); wcomulada@mednet.ucla.edu (W.S.C.); sojimenez@mednet.ucla.edu (S.J.); mrotheram@mednet.ucla.edu (M.J.R.-B.); 2 School of Health Sciences, Merrimack College, North Andover, MA 01845, USA; nsantabarbara@gmail.com; 3 Section of Adolescent Medicine, Department of Pediatrics, School of Medicine, Tulane University, New Orleans, LA 70112, USA; mocasio@tulane.edu; 4 Department of Family and Community Medicine, University of Texas Southwestern Medical Center, Dallas, TX 75390, USA; liz.arnold@utsouthwestern.edu; 5 Department of Pediatrics, David Geffen School of Medicine, University of California Los Angeles, Los Angeles, CA 90095, USA; knielsen@mednet.ucla.edu; 6 Department of Public Health, College of Osteopathic Medicine, Nova Southeastern University, Fort Lauderdale, FL 33328, USA; mariafer@nsu.nova.edu

**Keywords:** COVID-19, youth, attitudes, prevention behaviors, HIV, gay/bisexual, transgender

## Abstract

Sexual and gender minority (SGM) and racial or ethnic minority youth at-risk for or living with HIV may have higher risk of SARS-CoV-2 infection. However, there are few data on vaccine hesitancy/acceptance and COVID-19 self-protective behaviors among this population. Youth aged 15–24 years (*n* = 440), predominantly African American and Latine (73%, *n* = 320) SGM, from Los Angeles and New Orleans reported their vaccine attitudes and COVID-19 and HIV preventive behaviors in October 2020. Latent class analyses categorized individuals into groups based on their vaccine attitudes and preventive behaviors. Relationships between these groups and other factors were analyzed using Fisher’s exact tests, ANOVA, and logistic regression. Most youth had accepting vaccine attitudes (70.2%, *n* = 309), with 20.7% hesitant (*n* = 91), and 9.1% resistant (*n* = 40). SGM and African Americans were significantly less accepting than their cis-gender and heterosexual peers. About two-thirds (63.2%, *n* = 278) of the respondents reported consistent COVID-19 self-protective behaviors. Youth with pro-vaccine attitudes were most consistently self-protective; however, only 54.4% (*n*= 168/309) intended to take a COVID-19 vaccine. Homelessness history, race, and sexual orientation were associated with vaccine attitudes. Accepting vaccine attitudes and consistent COVID-19 self-protective behaviors were closely related. COVID-19 attitudes/behaviors were not associated with HIV risk and only loosely associated with SARS-CoV-2 vaccine intentions.

## 1. Introduction

Sexual and gender minority (SGM) and racial or ethnic minority young people, such as Black and Latine, gay, bisexual, transgender, and non-binary people, are at increased risk of acquiring and dying from COVID-19 [1], in addition to HIV [2]. However, there are few data and almost no surveillance on COVID-19 risks among SGM young people [3,4]. SGM youth experience various stressors and forms of stigma and discrimination, which may impact health outcomes [5]. For example, SGM youth experience high rates of family rejection based on their sexual orientation or gender identity, which, coupled with other risk factors, result in a high risk of several related factors, such as homelessness, mental health disorders, suicidal ideations and attempts, hospitalizations, and alcohol and drug abuse [6,7,8]. The stigmatizing and discriminatory stressors of being SGM may also be elevated among Black and Latine SGM youth [9,10]. Furthermore, evidence indicates that Black and Latine Americans experience a disproportionate burden of COVID-19 morbidity and mortality and face structural inequalities that contribute to these disparities [11,12,13,14]. For COVID-19 vaccines, in Black, Indigenous, and People of Color (BIPOC) communities, hesitancy may be a form of protection given historical experiences with racism and research that have led to a lack of trust [15].

Given youth’s histories of risk, it is unclear how young people will respond to a threat, such as COVID-19. Potentially, the increased risk of acquiring HIV may cause racial or ethnic minority and SGM youth to be more health protective than their peers. Alternatively, youth consistently put at risk may underestimate their likelihood of acquiring COVID-19; repeated experiences of risky situations can result in an underestimation of the likelihood of an event, in particular for rarer events, a phenomenon known as the description–experience gap [16]. In individuals struggling to survive on a daily basis, the risk of disease acquisition can often appear remote [17,18]. Moreover, as younger people are less likely to experience serious illness from a COVID-19 infection [19], it is unclear whether prosocial motivations (e.g., to prevent infection among more vulnerable individuals) are sufficient to compel youth to engage in risk-reduction behaviors.

This study examines both general vaccination attitudes, SARS-CoV-2 vaccination intentions, and COVID-19 self-protective behaviors (i.e., mask-wearing, social distancing, hand washing, and isolating) recommended to prevent COVID-19 infection among young people at risk for or living with HIV. A survey was conducted in the context of ongoing HIV intervention trials among young people aged 18–26 in Los Angeles, California, and New Orleans, Louisiana [20,21]. These ongoing studies enabled contextual comparisons from a large city and county on the West Coast and another geographically smaller city in the South—factors having already been associated with vaccine acceptance, hesitancy, and resistance [22]. Prior research identified racial or ethnic disparities in HIV Pre-Exposure Prophylaxis (PrEP) use in New Orleans, with lower rates of use among Black compared to White youth, but no differences in Los Angeles [23]. In addition, a large percentage of the study samples were African American and Latine, and with histories of homelessness—factors associated with hesitant or resistant attituded to COVID-19 vaccines [22,24].

## 2. Materials and Methods

All study procedures were reviewed and approved by the institutional review boards of the University of California, Los Angeles (UCLA IRB#16-001372) and Tulane University Review Board (Tulane IRB#1033876).

### 2.1. Participants

SGM youth and those with histories of homelessness, mental health hospitalizations, substance abuse treatment, incarceration, sex work or transactional sex, sexually transmitted infections (STIs), HIV, and condomless anal sex in the prior 12 months were recruited from 13 community-based agencies, clinic sites, and social networking apps in Los Angeles and New Orleans for three HIV intervention protocols from 2017–2019 [25]. Young people aged 12–24 years old were enrolled and reassessed at four-month intervals for 24 months. In October 2020, participants were invited to complete an assessment of their COVID-19 related experiences, attitudes, and behaviors in anticipation of vaccine availability, surges in COVID-19 infections, lockdowns, and remote learning. Since this was an exploratory study embedded in an ongoing group of cohort studies, sample size calculations were not conducted. A total of 440 young people aged 15–24 years old (*n* = 52 youth living with HIV) completed online questionnaires of 483 that responded with incomplete responses, of the 1716 previously enrolled study participants that were invited to participate.

### 2.2. Assessments

A Qualtrics survey link was sent to study participants via text messages and/or emails with unique web links to the survey throughout October 2020. The survey took 15–20 min to complete and the participants were compensated $25. In addition to questions about the vaccine, we collected data on background and demographics, including their lifetime experiences related to HIV risk. This paper examines three primary measures from the survey.

#### 2.2.1. Vaccine Attitudes

General vaccine attitudes were evaluated using 8 items adapted from the Vaccine Hesitancy Scale (VHS) developed by the World Health Organization (WHO) SAGE Working Group for Vaccine Hesitancy [26]. The VHS scale assesses agreement on the effectiveness, trustworthiness, and safety of childhood vaccines on a 5-point Likert scale among parents. Questions cover whether vaccines were important to one’s health, effective, protect against disease, important to community health, and beneficial, as well as trust in public health officials, worry about side effects, and riskiness (See the Supplementary Materials for the adapted scale questions).

#### 2.2.2. COVID-19 Protective Behaviors

Four behaviors to mitigate the risk of COVID-19 transmission/acquisition were self-reported: social distancing, isolation, mask wearing, and handwashing. Each item was reported using a checklist format (yes/no) for two time periods: (1) the main ‘lock-down’ period of around 20 March to the middle of May, and (2) the summer re-opening period from June to October 2020 (before the winter surge of 2020–2021). For the latent class analysis, responses were clustered as: Beginning (March–May 2020), Later (May 2020–time of survey), or Always/Adherent (March 2020–time of survey).

#### 2.2.3. SARS-CoV-2 Vaccination Intentions

The likelihood of vaccinating (1) or not (0) was assessed, “What is the likelihood that you will get a COVID-19 vaccination when it is available?”, on a 5-level scale (‘Very likely’, ‘Likely’, ‘Somewhat likely’, ‘Not likely’, or ‘Refuse to answer/don’t know’). We dichotomized responses into intention (‘Likely’ and ‘Very likely’) and low intention (all other answers).

### 2.3. Data Analyses

We used Latent Class Analysis (LCA) to categorize individuals into specific groups based on vaccine attitudes and COVID-19 self-protective behaviors. Given a fixed number of classes, LCA uses different variables to find natural groupings in the data, which then estimates how likely each individual fits into each class. Each participant’s predicted class is based on the similarity of their responses to the class. For both the vaccine attitudes and the self-protective behavior classes, we chose the number of classes based on goodness of fit metrics (e.g., BIC) and interpretability. We used the poLCA package in R to fit the LCA models.

Vaccine attitudes were best reflected in three latent classes. We interpreted the groupings as accepting, hesitant, and resistant (the distribution of responses to vaccine attitude questions across the three latent classes are presented in Supplementary Figure S1). Two latent classes for self-protective behaviors provided a sensible grouping: adherent and inconsistent (the distribution of responses across the two latent classes are presented in Supplementary Figure S2).

For both predicted attitudinal and behavioral classes, we first examined how sociodemographic and personal risk histories may or may not be associated with the classes using contingency tables and Fisher’s exact tests for categorical variables and sample means and ANOVA for numeric variables. Using the significantly related factors, we first fit a multinomial logistic regression for attitudes. This model essentially fits a resistant versus hesitant model and a resistant versus accepting model; however, we found little difference between the resistant and hesitant classes. Therefore, we fit an accepting versus not accepting logistic regression model, which is also easier to interpret than a multinomial outcome; this was the primary model for evaluating vaccine attitudes (results of the multinomial model are available in Supplementary Table S1). Using the same predictors as the vaccine attitudes model, we fit an additional logistic regression model for intention to receive a COIVD-19 vaccine when it became available, which examined whether the same factors predicted both vaccine attitudes and intentions to take a not-yet-available COVID-19 vaccine. Finally, we fit another logistic regression model for the self-protective behavior classes using the significantly related factors.

## 3. Results

### 3.1. Sample

As shown in Table 1, the sample was largely African American (39.2%, *n* = 173) and Latine (33.4%, *n* = 147), with 16.6% (*n* = 73) white and 10.7% (*n* = 47) of other ethnicities. About two-thirds of the sample was recruited in Los Angeles (*n* = 303) and one-third in New Orleans (*n* = 137). The sample was predominantly cis-male (76.9%, *n* = 325)), gay, or bisexual (74.1%, *n* = 326), with a mean age of 23 years old. A minority (11.8%, *n* = 52) of young people were living with HIV. Most youth had health insurance (80.5%, *n* = 354), about half were employed, and 31.8% (*n* = 140) reported hard drug use. About 21% (*n* = 94) reported a prior hospitalization for mental illness, 13% (*n* = 59) had been in substance abuse-treatment programs, 11.7% (*n* = 51) had been incarcerated, and 34.8% (*n* = 153) had been homeless. At enrollment, about 61% (*n* = 272) of the youth reported unprotected anal sex in the last year, 79.1% (*n* = 348) of youth reported not using condoms all the time and did not consistently use condoms, and only 14% (*n* = 62) were using PrEP to protect themselves from HIV.

### 3.2. Attitudes

Table 1 compares the sociodemographic characteristics and risk histories of youth classed as vaccine resistant, hesitant, and accepting. Overall, 9% (*n* = 40) were resistant, 20% (*n* = 91) hesitant, and 70% accepting (*n* = 309). The univariate analysis (see Table 1) found that race/ethnicity, gender, sexual orientation, age, monthly income, participation in a substance abuse treatment program, previous hospitalization for mental health, previous incarceration, previous homelessness, and intention to take a COVID-19 vaccine were significantly (*p* < 0.05) associated with vaccine attitudes. There were no differences based on HIV-related factors (i.e., condom use, PrEP/PEP use, or HIV status). In the multivariate logistic regression model (see Table 2) comparing the accepting group to others, we found that only race/ethnicity, sexual orientation, and homelessness were significantly associated with vaccine acceptance. We estimated that youth of all other ethnicities had higher odds of having more accepting vaccine attitudes than African American youth, with white individuals having the highest odds in comparison. Relative to gay youth, pansexual/asexual/other youth had significantly greater odds of vaccine acceptance; we did not observe significant differences in the odds of vaccine acceptance between bisexual and gay or heterosexual participants. Finally, the odds of vaccine acceptance were significantly lower among those who had experienced homelessness relative to those who had not experienced homelessness.

### 3.3. COVID-19 Prevention Behavior

Table 1 also compares the sociodemographic characteristics and risk histories of youth classed as inconsistent and adherent to COVID-19 self-protective behaviors. We found that 36.8% (*n* = 162) were inconsistent and 63.2% (*n* = 278) were adherent. Univariate analysis indicated that race/ethnicity, gender, monthly income, previous hospitalization for mental health, previous incarceration, previous homelessness, and intention to take a COVID-19 vaccine were significantly associated with prevention behaviors. There were no differences based on HIV-related factors (i.e., condom use, PrEP/PEP use, or HIV status). In the multivariate logistic regression model, only race/ethnicity and homelessness were associated with prevention behaviors (see Table 2). The odds of adherent behaviors were significantly lower among African Americans relative to White and Latine individuals. Moreover, the odds of adherent behaviors were significantly lower among those who had experienced homelessness relative to those who had not experienced homelessness. We estimated that all ethnicities had higher odds of being adherent than African Americans (Latine, White, and others had similar odds). Youth with lifetime homelessness experiences also had lower odds of prevention adherence.

### 3.4. SARS-CoV-2 Vaccine Intentions

Table 2 also summarizes the results of the multivariate logistic regression analyses examining the predictors of the intention to take a COVID-19 vaccine when available. Only race/ethnicity and gender were associated with intention. The odds of intending to receive a COVID-19 vaccine were lower among African Americans relative to White individuals. Latine and African American individuals had comparable odds of intending to receive a COVID-19 vaccine. Relative to Cis-males, Cis-females exhibited lower odds of intending to receive a COVID-19 vaccine. We did not observe any other differences in the odds of intending to receive a COVID-19 vaccine between Cis-males and any other gender group.

### 3.5. Comparison between Attitudes, Behaviors, and Intentions

**Figure 1:**
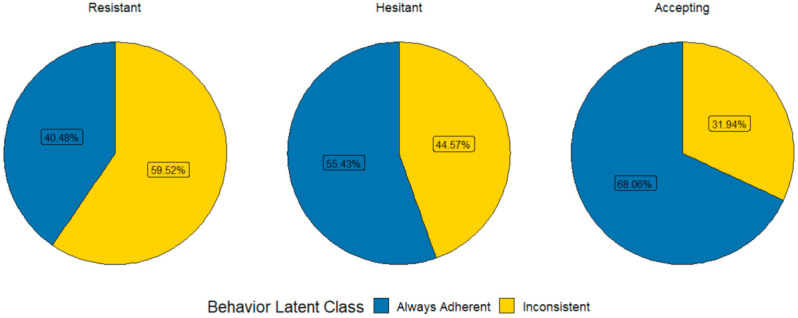
Comparisons of COVID-19 prevention behavior classes by vaccine attitude classes.

Figure 1 shows a breakdown of the protective behaviors for each vaccine class. About 40% (*n* = 16/40) of participants with resistant attitudes were adherent to self-protective behaviors, compared to 55.4% (*n* = 50/91) for the hesitant and 68.1% (*n* = 210/309) for the accepting groups. General vaccine attitudes were associated with self-protective behaviors, and intention to receive a COVID-19 vaccine was associated with both the attitude and behavioral classes. Of those who intended to receive a COVID-19 vaccine, 88.9% fell into the pro-vaccine group (*n* = 168/189), and 69.8% (*n* = 132/189) fell into the self-protective group. However, only 54.4% (*n*= 168/309) of the pro-vaccine group and 47.5% (*n* = 132/278) of the adherent group intended to receive a COVID-19 vaccine.

## 4. Discussion

The rates of general vaccine attitudes reflecting acceptance, hesitancy, and resistance observed in our study participants are highly similar to those of young peoples’ attitudes documented both domestically [22] and globally [27]. Similarities were noted when comparing responses regarding vaccine attitudes from youth nationally or specifically in the Southern U.S. [28]—the site where one-third of our study participants resided at the time of the survey. Similar to what is described in national estimates [22], African American young people and SGM youth are also more likely to have hesitant and resistant attitudes towards COVID-19 vaccination and self-protective behaviors. However, Stephenson et al. [29] found contradictory results—black/African Americans in their study of gay, bisexual, and other MSM were more likely to report willingness to accept vaccination.

The close relationship between vaccine attitudes and prevention behaviors found here is less commonly documented in the literature. Even when examining COVID-19 attitudes and behaviors, other researchers [30] found larger gaps between attitudes, preventive behaviors, and intentions. It may be that the young people in this study, on the topic of COVID-19 generally, were less polarized than adults or had fewer discrepancies between their attitudes, intentions, and behaviors.

It is noteworthy that COVID-19 vaccine intention was lower than general vaccine accepting attitudes. Open-ended responses provided by some participants indicated concerns about the rapidity of vaccine development and mistrust of the presidential administration, rather than vaccines more generally. More broadly, in a recent study, acceptance or positive attitudes about getting a COVID-19 vaccine predicted vaccination intention [31]. As vaccine rollout progressed and vaccine availability increase, normative pressure may increase as more people get vaccinated. However, normative pressure and vaccine intentions are still lower among the Black community of all ages compared to the White and Latine communities due to institutional mistrust and structural racism [32]. Vaccine hesitancy among members of the Black community can be related to historical mistreatment and other factors, such as an accelerated vaccine timeline, the government’s response to the pandemic, and the lack of transparency of the scientific process involved in the development and approval of COVID-19 vaccines [33,34].

Similarly, vaccine hesitancy among LGBTQ+ populations has been attributed not only to disparities in access to healthcare systems, but also mistrust in healthcare professionals and government information, which is also rooted in historical marginalization and stigmatization [4]. One global survey of COVID-19 vaccine acceptance by Lazarus et al. collected data in June of 2020, 4 months prior to the collection of data presented here, found that vaccine acceptance varied by country, ranging from 89% in China to 55% in Russia, with the United States in the upper-middle range of 75%. Notably, in this sample, 70% (*n* = 309) of youth reported intention to vaccinate for SARS-CoV-2 when a vaccine became available, which is more in line with the data from the United Kingdom (72%), Italy (71%), and Canada (69%) presented by Lazarus et al. [35]

Notably, there was no association observed in this study between HIV-related factors (e.g., condom use, PrEP/PEP use, or HIV status) and vaccine attitudes and COVID-19 prevention behaviors. For youth living with HIV and youth highly engaged in HIV prevention, vaccine acceptance and engaging in protective behaviors for COVID-19 may be related to lessons gleaned from understanding the importance of behavior change and adaptation in response to the HIV pandemic. This might include dynamics of stigma related to an illness and strategies to cope with it, the ways public health can be politicized, and promises of vaccines [36]. Black and Latine PLWH in a recent study noted being early adopters of recommended COVID-19 prevention behaviors and described themselves as more adherent to recommendations than their peers in the larger community, even though they experienced structural barriers and medical mistrust while navigating a pandemic that once again disproportionately affected communities of color [37].

Young people in this study with life histories with the most significant risks, specifically homelessness, were associated with more negative attitudes and behaviors, as well as lower intention to vaccinate. The unique life history of these youth and the associated risk profiles suggested the need for tailored outreach efforts and programs to address their needs and encourage vaccine uptake [4]. This is especially relevant considering that the youth identified here having low intention to vaccinate may have different reasons for doing so than others who are vaccine hesitant. For example, a systematic review on COVID-19 vaccine hesitancy in LGBTQ+ populations by Garg et al. discussed the utility of the epidemiologic triad approach to understanding vaccine hesitancy. This model includes environmental, agent, and host factors as important to consider when evaluating vaccine hesitance/acceptance in a population. Youth at risk for and living with HIV may experience unique factors at each of these levels that could impact their vaccine acceptance (or lack thereof). For example, at the environmental level, stigmatizing policies may reduce trust in governmental, medical, or public health sources of information related to vaccine rollout. Similarly, perceived disease susceptibility may be influenced by one’s own history of illness, such as HIV (agent factors), and one’s income level (potentially influenced by past homelessness or incarceration) may influence one’s ability to access a trusted medical professional (host factors) [4].

These results suggest the need to better understand the underlying causes of low vaccine uptake in order to appropriately tailor education campaigns and vaccination efforts for SARS-CoV-2 as well as HPV and potentially HIV. It is notable that only homelessness emerges in multivariate analyses as a predictor of self-protective behaviors, which may parallel trends observed in HIV prevention, in which people who experience homelessness or other forms of socioeconomic marginalization have lower levels of HIV preventive behaviors, reflecting more proximal hierarchies of needs (i.e., shelter, food, or income) compared to risks of HIV or COVID-19 infection and disease. Together, these results suggest the importance of targeting youth with histories of institutionalization and homelessness regarding COVID-19 vaccinations. Because of the relative recency of COVID-19, it is unclear whether public health departments are following the general approach adopted for HPV vaccinations with young people—to address parents [38]. The young people in this sample were generally young adults; thus our results highlight the importance of targeting youth themselves, not parents, in order to achieve high vaccination rates.

### Limitations

The predicted attitudinal and behavioral classes were estimates from data with inherent uncertainty, so all inferences involving these predicted classes should be reported with some caution. These data were collected just prior to the availability of COVID-19 vaccines and before major surges in hospitalizations starting in December 2020; therefore, it is critical to follow-up with these young people as their intentions at the time of the survey may not be consistent with their later vaccination decisions. Follow-up data on current vaccination status and attitudes are currently being collected and will be valuable in understanding how youth’s beliefs and attitudes may have changed over time. Furthermore, the results of this study may not be generalizable to all SGM or racial or ethnic minority youth in the U.S., as the participants were recruited from youth-serving community-based organizations and clinics, and social networking/dating apps in Los Angeles and New Orleans; however, the results maybe be reflective of youth encountered in similar settings.

## 5. Conclusions

Considering the rapid rise of COVID-19 cases in the US with the spread of new variants, vaccinations, including boosters, have proven to be critical not only in preventing the spread of disease, but also in preventing severe morbidity and mortality [39]. Although these data do not include whether participants are currently vaccinated, those identified here as having low intentions to vaccinate may now be at even greater risk of becoming infected, developing severe symptoms, and potentially requiring hospitalization. Considering the unique needs of these youth, broader population-based approaches, such as generalized advertisement campaigns or state-sponsored lottery systems, may fail to adequately address this population’s underlying doubts that contribute to their vaccine hesitancy and/or resistance. Furthermore, vaccination campaigns targeting youth may fail to reach members of this population who expressed hesitance or resistance to COVID-19 vaccination. This may be especially relevant for youth experiencing homelessness or who have experienced other risk factors identified here as significant predictors of COVID-19 vaccine intentions. As such, public health efforts to increase vaccination rates among this population may benefit from a more targeted approach, such as utilizing community leaders to better understand and address existing doubts about vaccine safety/efficacy, purposefully utilizing non-stigmatizing language and imagery in health communication campaigns, and utilizing existing networks of community-based organizations to target outreach efforts. Such programs may address the need for targeted outreach programs to address vaccine hesitancy and resistance among vulnerable youth, particularly those living with HIV and those with experiences of substance abuse treatment, mental health hospitalization, incarceration, or homelessness.

## Figures and Tables

**Table 1:** ^1^ General vaccine attitudes and COVID-19 protective behavior classes among youth at-risk for or living with HIV in Los Angeles and New Orleans.

	General Vaccine Attitude			COVID-19 Prevention Behavior		
	Resistant	Hesitant	Accepting	Inconsistent	Adherent	Total
	40 (9.1%)	91 (20.7%)	309 (70.2%)	162 (36.8%)	278 (63.2%)	440
**Socio-Demographic Factors**						
**Ethnicity**	*p* < 0.001 ***			*p* < 0.001 ***		
African Amer.	25 (14.5%)	56 (32.4%)	92 (53.2%)	85 (49.1%)	88 (50.9%)	173 (39.2%)
Latine	10 (6.8%)	24 (16.3%)	113 (76.9%)	44 (29.9%)	103 (70.1%)	147 (33.4%)
Other	2 (4.3%)	5 (10.6%)	40 (85.1%)	13 (27.7%)	34 (72.3%)	47 (10.7%)
White	3 (4.1%)	6 (8.2%)	64 (87.7)	20 (27.4%)	54 (72.6%)	73 (16.59%)
**Location**	*p* = 0.601			*p* = 0.169		
Los Angeles	25 (8.3%)	62 (20.5%)	216 (71.2%)	105 (34.7%)	198 (65.4%)	303 (68.9%)
New Orleans	15 (11.0%)	29 (21.2%)	93 (67.9%)	57 (41.6%)	80 (58.39%)	137 (31.1%)
**Gender**	*p* < 0.001 ***			*p* < 0.001 ***		
Cis-Man	27 (8.3%)	52 (16.0%)	246 (75.7%)	115 (35.4%)	210 (64.6%)	325 (73.9%)
Cis-Woman	8 (16.7%)	22 (45.8%)	18 (37.5%)	23 (47.9%)	25 (52.1%)	48 (10.9%)
Non-binary	1 (3.7%)	8 (29.6%)	18 (66.7%)	9 (33.3%)	18 (66.7%)	27 (6.1%)
Trans Fem.	1 (5.0%)	5 (25.0%)	14 (70.0%)	9 (45.0%)	11 (55.0%)	20 (4.6%)
Trans Masc.	3 (15.0%)	4 (20.0%)	13 (65.0%)	3 (30.0%)	14 (70.0%)	20 (4.6%)
**Sexual Orient.**	*p* < 0.001 ***			*p* = 0.419		
Gay	13 (5.6%)	37 (16.0%)	182 (78.5%)	83 (35.8%)	149 (64.2%)	232 (52.7%)
Bisexual	12 (12.8%)	25 (26.6%)	57 (60.6%)	36 (38.3%)	58 (61.7%)	94 (21.4%)
Pansexual/Asexual/Other	3 (5.4%)	8 (14.3%)	45 (80.4%)	13 (32.2%)	43 (76.8%)	56 (13.7%)
Heterosexual	12 (20.7%)	21 (36.2%)	25 (43.1%)	30 (51.7%)	23 (48.3%)	58 (13.2%)
**Age**	*p* = 0.028 *			*p* = 0.284		
Mean (SD)	23.2 (2.5)	24.0 (2.1)	23.3 (2.2)	23.6 (2.4)	23.3 (2.2)	23.4 (2.2)
**Employment**	*p* = 0.133			*p* = 0.278		
Yes (Full or Part Time)	18 (7.9%)	40 (17.6%)	169 (74.5%)	78 (34.4%)	149 (65.6%)	227 (51.6%)
No/Refused/DK	22 (1.3%)	51 (23.9%)	140 (65.7%)	84 (39.4%)	129 (60.6%)	213 (48.4%)
**Monthly Income ($)**	*p* = 0.022 *			*p* = 0.007 **		
Mean (SD)	901.4 (1053.9)	821.1 (1020.1)	1353.4 (1674.8)	1372.1 (1627.1)	966.1 (1291.2)	1222.9 (1523.2)
**Health Insurance**	*p* = 0.139			*p* = 0.278		
Yes	28 (7.9%)	71 (20.1%)	255 (72.0%)	78 (34.4%)	149 (65.6%)	354 (80.5%)
No/Refused/DK	12 (9.3%)	20 (23.3%)	54 (62.8%)	84 (39.4%)	129 (60.6%)	86 (19.6%)
**Risk Factors**						
**HIV Status ^**	*p* = 0.171			*p* = 0.284		
Positive	4 (7.7%)	16 (30.8%)	32 (61.5%)	23 (44.2%)	29 (55.8%)	52 (11.8%)
Negative	36 (9.3%)	75 (19.3%)	277 (71.4%)	139 (35.8%)	249 (64.2%)	388 (88.2%)
**Drug Use Lifetime (self-reported) ^2^**	*p* = 0.794			*p* = 0.598		
Yes	11 (7.9%)	28 (20.0%)	101 (72.1%)	49 (35.0%)	91 (65.0%)	140 (31.8%)
No	29 (9.7%)	63 (21.0%)	208 (69.3%)	113 (37.7%)	187 (62.3%)	300 (68.2%)
**Ever been in Substance Abuse Program**	*p* = 0.049 *			*p* = 1.000		
Yes	10 (16.9%)	14 (23.7%)	35 (59.3%)	22 (37.3%)	37 (62.7%)	59 (13.4%)
No	30 (7.9%)	77 (20.2%)	274 (71.9%)	140 (36.7%)	241 (63.3%)	381 (86.6%)
	**General Vaccine Attitude**			**COVID-19 Prevention Behavior**		
	**Resistant**	**Hesitant**	**Accepting**	**Inconsistent**	**Adherent**	**Total**
**Ever Hospitalized for Mental Health**	*p* = 0.018 *			*p* = 0.030 *		
Yes	13 (13.8%)	26 (27.7%)	55 (58.5%)	44 (46.8%)	50 (53.2%)	94 (21.4%)
No	27 (7.8%)	65 (18.8%)	254 (73.4%0	118 (34.1%)	228 (65.9%)	346 (78.6%)
**Previous Incarceration ^**	*p* = 0.001 **			*p* = 0.031		
Yes	8 (15.7%)	18 (35.3%)	25 (49.0%)	26 (51.0%)	25 (49.0%)	51 (88.3%)
No	32 (8.3%)	70 (18.1%)	284 (73.6%)	135 (35.0%)	251 (65.0%)	386 (11.7%)
**Condomless Anal Sex Past Year ^**	*p* = 0.572			*p* = 1.000		
Yes	22 (8.1%)	55 (20.2%)	195 (71.7%)	101 (37.1%)	171 (62.9%)	272 (62.1%)
No	18 (10.8%)	35 (21.1%)	113 (68.1%)	61 (36.7%)	105 (63.3%)	166 (37.9%)
**Ever Homeless**	*p* < 0.001 ***				*p* < 0.001 ***	
Yes	21 (13.7%)	53 (34.6%)	79 (51.6%)	78 (49.0%)	75 (51.0%)	153 (34.8%)
No	19 (6.6%)	38 (13.2%)	230 (80.1%)	84 (29.3%)	203 (70.7%)	287 (65.2%)
**Condomless Sex w/HIV+**	*p* = 0.888			*p* = 0.798		
Yes	3 (6.7%)	11 (24.4%)	31 (68.9%)	16 (35.6%)	29 (64.4%)	45 (10.2%)
No	34 (9.3%)	73 (19.9%)	259 (70.8%)	137 (37.4%)	229 (62.6%)	388 (83.2%)
Refused/NA	3 (10.3%)	7 (24.1%)	19 (65.5%)	16 (31.0%)	20 (69.0%)	29 (6.6%)
**PrEP/PEP use at Enrollment**	*p* = 0.169			*p* = 0.395		
Yes	3 (4.8%)	9 (14.5%)	50 (80.6%)	26 (41.9%)	36 (58.1%)	62 (14.1%)
No/DK/Missing	37 (9.8%)	82 (21.7%)	259 (68.5)	136 (36.0%)	242 (64.0%)	378 (85.9%)
**Condom Use at Enrollment**	*p* = 0.215			*p* = 0.483		
Always	5 (5.4%)	14 (15.2%)	73 (79.3%)	35 (38.0%)	57 (62.0%)	92 (20.9%)
Sometimes	16 (8.1%)	41 (20.7%)	141 (71.2%)	79 (39.9%)	119 (60.1%)	198 (45.0%)
Never	11 (13.1%)	21 (25.0%)	52 (61.9%)	28 (33.3%)	56 (66.7%)	84 (19.1%)
Refused/NA	8 (12.1%)	15 (22.7%)	43 (71.2%)	20 (30.3%)	46 (69.7%)	66 (15.0%)
**COVID-19 Vaccine Intent**	*p* < 0.001 ***			*p* = 0.013 **		
High	7 (3.7%)	14 (7.4%)	168 (88.9%)	57 (30.2%)	132 (69.8%)	189 (43.0%)
Low	33 (13.1%)	77 (30.7%)	141 (56.2%)	104 (41.8%)	146 (58.2%)	251 (57.1%)

^1^, *** indicates *p* < 0.001, ** *p* < 0.01, and * *p* < 0.05. ^ indicates some missing values. ^2^ Excluding Cannabis due to high proportion reporting use.

**Table 2:** Multivariate logistic regression estimates and 95% confidence intervals (Odds-Scale) for models examining predictors of general vaccine attitudes, SARS-CoV-2 vaccine intentions, and COVID-19 prevention behaviors among youth at-risk for and living with HIV in Los Angeles and New Orleans.

	Response		
Factor	General Vaccine Attitude (Accepting vs. Not)	SARS-CoV-2 Vaccine Intention (High vs. Low)	COVID-19 Prevention Behaviors (Adherent vs. Inconsistent)
**Race (ref. African American)**			
Latine	2.555 (1.492, 4.444) *	1.414 (0.866, 2.315)	2.136 (1.319, 3.488) *
Other	4.762 (1.957, 13.182) *	2.805 (1.409, 5.688) *	2.338 (1.145, 5.001) *
White	5.504 (2.408, 14.043) *	3.445 (1.863, 6.595) *	2.374 (1.276, 4.548) *
**Gender (ref. Cis-Male)**			
Cis-Fem	0.509 (0.229, 1.122)	0.336 (0.124, 0.813) *	1.083 (0.550, 2.146)
Non/Other	0.516 (0.229, 1.451)	0.609 (0.233, 1.500)	1.491 (0.623, 3.803)
Trans-Fem	0.966 (0.316, 3.206)	0.920 (0.317, 2.560)	0.794 (0.300, 2.163)
Trans-Male	0.310 (0.095, 1.046)	2.804 (0.971, 9.023)	1.393 (0.513, 4.249)
**Sexual Orientation (Ref. Gay)**			
Bisexual	0.671 (0.368, 1.237)	0.923 (0.537, 1.582)	n/a
Hetero	0.476 (0.217, 1.040)	0.762 (0.336, 1.684)	n/a
Pan/A/Other	2.539 (1.018, 6.877) *	1.008 (0.485, 2.088)	n/a
**Substance Abuse Treatment**	0.860 (0.409, 1.827)	0.844 (0.417, 1.688)	n/a
**Hospitalized for Mental Health**	0.869 (0.482, 1.585)	0.926 (0.524, 1.628)	0.774 (0.456, 1.319)
**Previous Incarceration**	0.998 (0.481, 2.086)	0.786 (0.363, 1.651)	0.931 (0.484, 1.797)
**Ever Homeless**	0.371 (0.214, 0.643) *	0.882 (0.526, 1.473)	0.486 (0.303, 0.777) *
**Monthly Income ($100)**	1.009 (0.009, 1.030)	1.003 (0.989, 1.017)	1.013 (0.997, 1.031)
**Age (Years)**	0.966 (0.861, 1.083)	1.027 (0.931, 1.134)	n/a

Note: We included significantly related factors from Table 1 as predictors for each response; sexual orientation, substance abuse, and age were not significant for behaviors, hence their exclusion. * indicates statistically significant results.

## Data Availability

Data will be uploaded to the DASH website as part of the NIH requirements for the parent study.

## Supplementary Materials

The following are available online at https://www.mdpi.com/article/10.3390/vaccines10030413/s1, Figure S1: Responses to vaccine attitude questions by vaccine latent class., Figure S2: Responses to COVID-19 prevention behavior questions by adherent and inconsistent latent classes. Table S1: Multinomial logistic regression results examining factors associated with resistant/hesitant vs. accepting and resistant vs. hesitant/acceptance.
