# Supplementary material for: Vaccine Attitudes and COVID-19 Vaccine Intentions and Prevention Behaviors among Young People At-Risk for and Living with HIV in Los Angeles and New Orleans
Source: Vaccines (Basel). 2022 Mar 9;10(3):413. doi: 10.3390/vaccines10030413 (PMC8954448; doi:10.3390/vaccines10030413)
Supplement: Supplementary file 1 [file vaccines-10-00413-s001.zip › vaccines-1585239-supplementary.pdf]

Supplementarly Materials

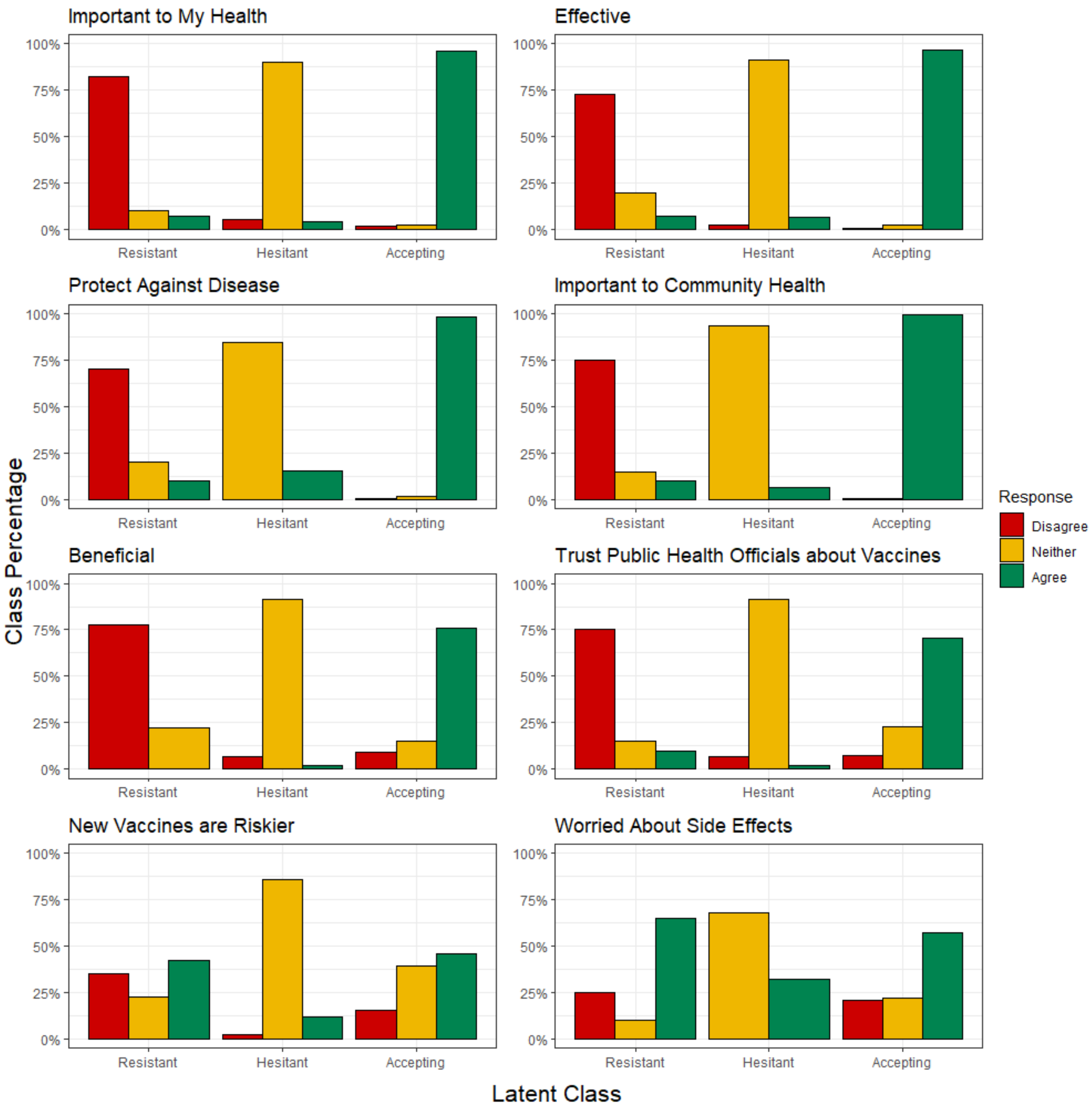

Figure S1. Responses to vaccine attitude questions by vaccine resistant, hesitant and accepting latent classes.

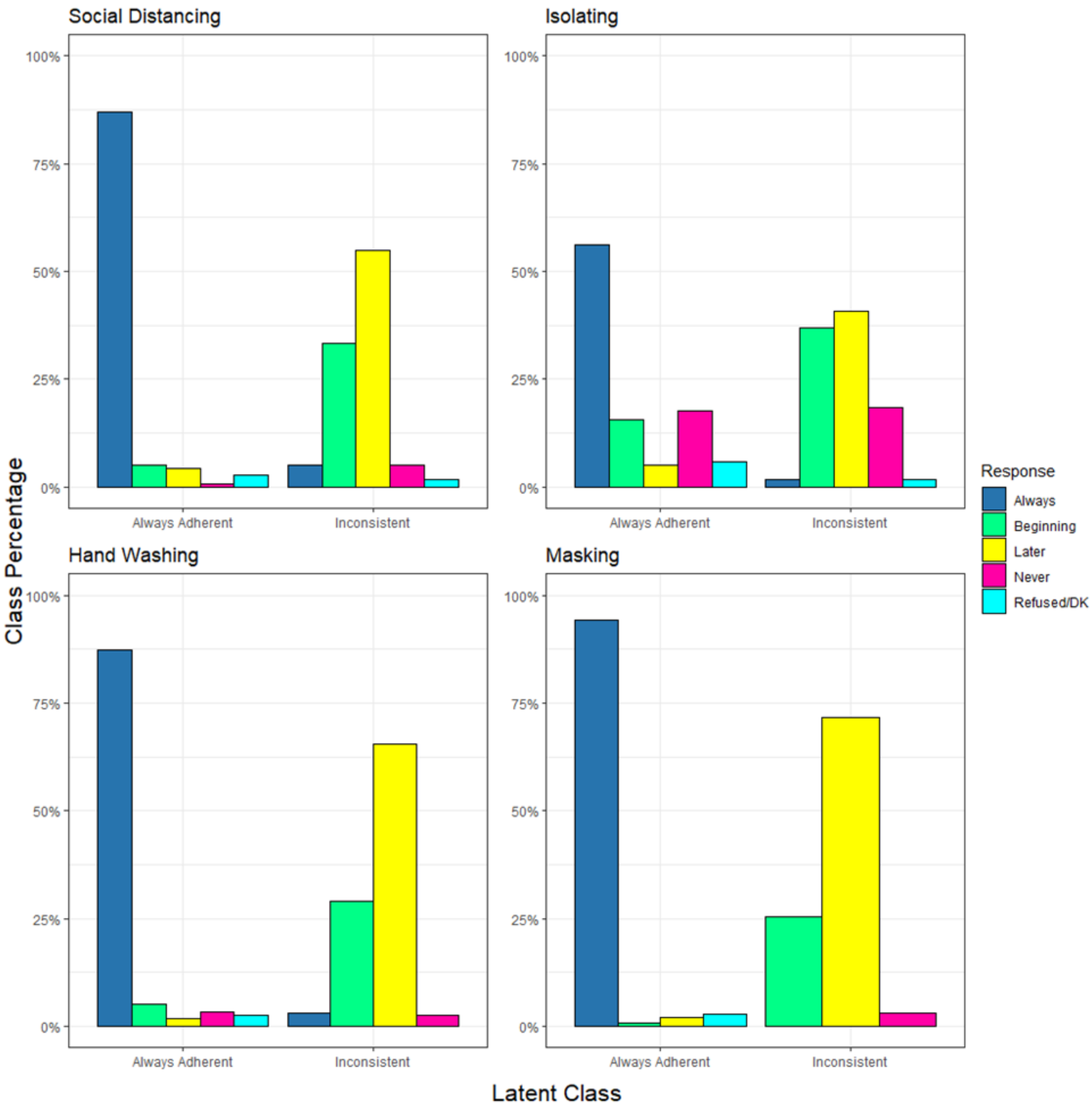

Figure S2. Responses to COVID-19 prevention behaviors questions by adherent and inconsistent latent classes.

Table S1. Multinomial logistic regression results examining factors associated with resistant/hesitant vs accepting and resistant vs hesitant/acceptance.

Factor	Estimate (95% CI)
**Model: Resistant + Hesitant**	
Intercept	0.010 (0.000, 0.794)
Latine	1.221 (0.475, 3.140)
Other	1.269 (0.215, 7.504)
White	1.059 (0.208, 5.391)
Cis-Women	2.728 (0.862, 8.633)
Nonconforming/Other	5.677 (0.594, 54.265)
Trans-Female	4.138 (0.404, 42.329)
Trans-Male	1.009 (0.161, 6.345)
Gay	1.424 (0.511, 3.964)
Hetero	0.506 (0.165, 1.551)
Pan/A/Queer/Other	0.943 (0.166, 5.352)
Substance Abuse Program	0.355 (0.115, 1.096)

Hospitalized for Mental Health	0.812 (0.322, 2.048)
Ever Incarcerated	1.362 (0.457, 4.060)
Homeless Lifetime	1.039 (0.413, 2.615)
Monthly Income ($100)	0.995 (0.960, 1.032)
Age (Years)	1.247 (1.030, 1.508)
**Model: Resistant + Accepting**	
Intercept	0.330 (0.007, 15.555)
Latino	2.919 (1.237, 6.887)
Other	5.538 (1.136, 27.000)
White	5.837 (1.460, 23.339)
Cis-Women	1.000 (0.321, 3.113)
Nonconforming/Other	1.994 (0.218, 18.226)
Trans-Female	2.667 (0.299, 23.787)
Trans-Male	0.312 (0.059, 1.644)
Gay	1.877 (0.746, 4.722)
Hetero	0.454 (0.156, 1.318)
Pan/A/Queer/Other	3.538 (0.730, 17.156)
Substance Abuse Program	0.442 (0.158, 1.235)
Hospitalized for Mental Health	0.751 (0.318, 1.774)
Ever Incarcerated	1.217 (0.421, 3.515)
Homeless Lifetime	0.382 (0.163, 0.893)
Monthly Income ($100)	1.005 (0.972, 1.038)
Age (Years)	1.121 (0.944, 1.332)
